# DFT Investigation of a Direct Z-Scheme Photocatalyst for Overall Water Splitting: Janus Ga_2_SSe/Bi_2_O_3_ Van Der Waals Heterojunction

**DOI:** 10.3390/ma18071648

**Published:** 2025-04-03

**Authors:** Fan Yang, Pascal Boulet, Marie-Christine Record

**Affiliations:** Aix-Marseille University, UFR Sciences, IM2NP, CNRS, 13013 Marseille, France; fan.yang.1@etu.univ-amu.fr (F.Y.); m-c.record@univ-amu.fr (M.-C.R.)

**Keywords:** 2D materials, hydrogen evolution reaction (HER), oxygen evolution reaction (OER), solar-to-hydrogen efficiency (STH), hydrostatic pressure, biaxial tensile strain, first-principles calculations

## Abstract

Constructing van der Waals heterojunctions with excellent properties has attracted considerable attention in the field of photocatalytic water splitting. In this study, four patterns, coined A, B, C, and D of Janus Ga_2_SSe/Bi_2_O_3_ van der Waals (vdW) heterojunctions with different stacking modes, were investigated using first-principles calculations. Their stability, electronic structure, and optical properties were analyzed in detail. Among these, patterns A and C heterojunctions demonstrate stable behavior and operate as direct Z-scheme photocatalysts, exhibiting band gaps of 1.83 eV and 1.62 eV. In addition, the suitable band edge positions make them effective for photocatalytic water decomposition. The built-in electric field across the heterojunction interface effectively inhibits electron-hole recombination, thereby improving the photocatalytic efficiency. The optical absorption coefficients show that patterns A and C heterojunctions exhibit higher light absorption intensity than Ga_2_SSe and Bi_2_O_3_ monolayers, spanning from the ultraviolet to visible range. Their corrected solar-to-hydrogen (STH) efficiencies are 13.60% and 12.08%, respectively. The application of hydrostatic pressure and biaxial tensile strain demonstrate distinct effects on photocatalytic performance: hydrostatic pressure preferentially enhances the hydrogen evolution reaction (HER), while biaxial tensile strain primarily improves the oxygen evolution reaction (OER). Furthermore, the heterojunctions exhibited enhanced optical absorption across the UV-visible spectrum with increasing hydrostatic pressure. Notably, a 1% tensile strain results in an improvement in visible light absorption efficiency. These results demonstrate that Ga_2_SSe/Bi_2_O_3_ heterojunctions hold great promise as direct Z-scheme photocatalysts for overall water splitting.

## 1. Introduction

The pursuit of renewable and eco-friendly energy solutions is essential in light of the growing global energy crises and environmental concerns [1,2]. The photocatalytic decomposition of water to produce hydrogen has emerged as a pivotal area of scientific research, owing to its potential to generate clean and renewable hydrogen energy [3,4,5]. However, several obstacles hinder the development of efficient photocatalysts, including appropriate band gap alignment [6,7], effective charge separation [8,9], and good chemical stability [9,10].

In the past few years, two-dimensional materials have gained significant attention in photocatalysis due to their distinctive physicochemical properties [11,12]. Among these, transition metal chalcogenides (TMCs) have emerged as particularly effective photocatalytic materials owing to their tunable band gaps [13,14], high carrier mobility [15,16], and substantial specific surface areas [15,17]. Metal oxides are also efficient photocatalysts for water decomposition to produce hydrogen, thanks to their excellent light absorption and electron-hole pair generation capabilities [18,19]. Gallium selenide sulfide (Ga_2_SSe), for example, demonstrates considerable promise for photocatalytic applications due to its unique electronic structure, which confers upon it remarkable visible light absorption properties [20]. R. da Silva et al. demonstrated that the Janus Ga_2_SSe monolayer has a stable structure, an indirect band gap, and low exciton binding energy. These features, along with efficient electron-hole separation and unique electronic alignment, make Ga_2_SSe highly suitable for photocatalytic water splitting and hydrogen generation [21]. Concurrently, bismuth oxide (Bi_2_O_3_), a well-established metal oxide photocatalyst, exhibits remarkable activity in photocatalytic reactions. P. Riente et al. explored the structure and electronic properties of α-Bi_2_O_3_ and β-Bi_2_O_3_ using first principles methods, and their findings aligned well with experimental data. Their photocatalytic activity arises from their narrowed band gaps, Bi 6s—O 2p hybridized orbitals, and red-shifted light absorption edges. Their optical properties highlight the potential of these polymorphs for efficient water splitting [22].

Single-component photocatalysts offer numerous beneficial properties. However, their performance is often hindered by a common issue: rapid photogenerated carrier recombination, which significantly limits photocatalytic efficiency [23]. To address this issue, designing heterojunctions is a promising approach to improving photocatalytic performance. By combining complementary materials, heterojunctions can effectively promote charge separation [24,25], inhibit recombination [26,27], and extend the range of light absorption [28,29]. Notably, van der Waals (vdW) heterojunctions, consisting of diverse two-dimensional (2D) materials, enhance photocatalytic water decomposition by improving interfacial interactions and overcoming the limitations of monolayer catalysts. R. Kumar et al. employed first principles methods to demonstrate that the C_2_N/WS_2_ vdW heterojunction exhibits improved photocatalytic performance for water splitting as a result of its type-II band alignment, efficient charge separation, and high visible light absorption. The heterojunction’s dual-layer mechanism facilitates both the oxidation and reduction of water, and thermodynamic analysis confirms its potential for efficient hydrogen generation. This study underscores the significant potential of heterojunction photocatalysis and can be a basis for developing next-generation 2D photocatalysts [30].

Heterojunctions, which can be directly employed in photocatalysis, include type-II and direct Z-scheme heterojunctions, both of which effectively separate photogenerated carriers and enhance redox reactions [31,32]. Van der Waals (vdW) heterojunctions consisting of 2D TMCs and metal oxides have shown significantly improved performance in photocatalytic water decomposition [13,33]. Recent research reveals that integrating 2D H-TiO_2_ with MoS_2_ or WS_2_ leads to the formation of vdW heterojunctions with enhanced stability, direct band gaps, and superior visible light absorption, bringing significant advancements in technologies related to photocatalytic and solar energy conversion [34]. The type-II ZnO/Ga_2_SSe and ZnO/GaSe heterostructures exhibit favorable bandgap characteristics and band edge alignments for photocatalytic water splitting. Notably, the ZnO/Ga_2_SSe heterostructure with sulfur vacancies demonstrates spontaneous hydrogen evolution reaction activity, achieving a substantial solar-to-hydrogen (STH) efficiency of 25.05% [35]. Similarly, the Z-scheme ZrS_2_/Ga_2_SSe van der Waals heterojunction, featuring an indirect bandgap of 1.33 eV, benefits from its optimized band structure and built-in electric field, which facilitate efficient electron transfer and significantly enhance photocatalytic performance. The heterojunction achieves an STH efficiency of 10.93% under a compressive strain of −6% [36]. The Bi_2_O_3_/MoS_2_ p-n heterojunction photocatalyst, with strong light-harvesting ability and efficient charge-carrier separation, exhibits superior visible-light-driven performance with a hydrogen conversion efficiency reaching up to 10 μmol h^−1^g^−1^ [37]. Additionally, the S-scheme TiO_2_/Bi_2_O_3_ heterojunction, with its optimized interface structure, reduces the migration distance of charge carriers, enhancing photocatalytic water-splitting performance for hydrogen production and displaying an H_2_ generation rate of 12.08 mmol h^−1^g^−1^ [38]. The systematic study of the electronic structure and photocatalytic performance of the various heterojunctions mentioned above provides valuable insights for guiding our research. This prompts an intriguing question: can we combine the high-performance Janus structure Ga_2_SSe with the metal oxide Bi_2_O_3_ to construct a novel Ga_2_SSe/Bi_2_O_3_ heterojunction for exploring its potential in photocatalysis?

In this work, the electronic properties, energy band alignment, and photocatalytic properties of the Ga_2_SSe/Bi_2_O_3_ heterojunction were systematically investigated using density-functional theory (DFT) approaches. The charge transfer within the heterojunction was analyzed through charge density difference and Bader charge calculations. To evaluate the photocatalytic potential of the heterojunction, its optical absorption properties and solar-to-hydrogen efficiency were also calculated. In parallel, we investigated the effects of hydrostatic pressure and biaxial strain on the photocatalytic performance of heterojunctions, focusing on their electronic properties and optical absorption characteristics. These results offer meaningful understanding of the photocatalytic mechanism of the Janus Ga_2_SSe/Bi_2_O_3_ heterojunction, highlighting the potential of this class of heterojunctions for hydrogen production through photocatalysis.

## 2. Computational Methods

In this study, all the calculations were carried out by means of density functional theory (DFT) [39,40] within the framework of the Vienna Ab initio Simulation Package (VASP) [41,42,43,44]. The Perdew–Burke–Ernzerhof (PBE) functional within the generalized gradient approximation (GGA) was utilized as an exchange-correlation functional [45], and the projector-augmented wave (PAW) method was employed to describe ion–electron interactions [46,47]. A plane-wave basis set was used with a cutoff energy of 450 eV and the first Brillouin zone was sampled with a k-point grid of 9 × 9 × 1. To minimize interactions between periodic images, a vacuum layer of 20 Å was implemented along the z-direction. Convergence thresholds were set to 10^−5^ eV for total energy and 0.05 eVÅ^−1^ for atomic forces, respectively. The DFT-D3 method proposed by Grimme [48] was utilized to account for van der Waals interactions. To accurately predict electronic structures, correct the underestimated band gaps by GGA functionals, and calculate optical properties, the Heyd–Scuseria–Ernzerhof (HSE06) hybrid functional, incorporating 20% exact exchange energy, was adopted [49,50]. Ab initio molecular dynamics (AIMD) simulations were conducted to assess thermal stability. The AIMD simulations, spanning 4 ps with a time step of 1 fs, were performed at a temperature of 300 K, regulated by the Nosé–Hoover thermostat. Additionally, Bader charge analysis [51] was performed to evaluate atomic charge distributions.

## 3. Results and Discussion

### 3.1. Structural Configurations and Stability of the Heterojunction

**Figure 1:**
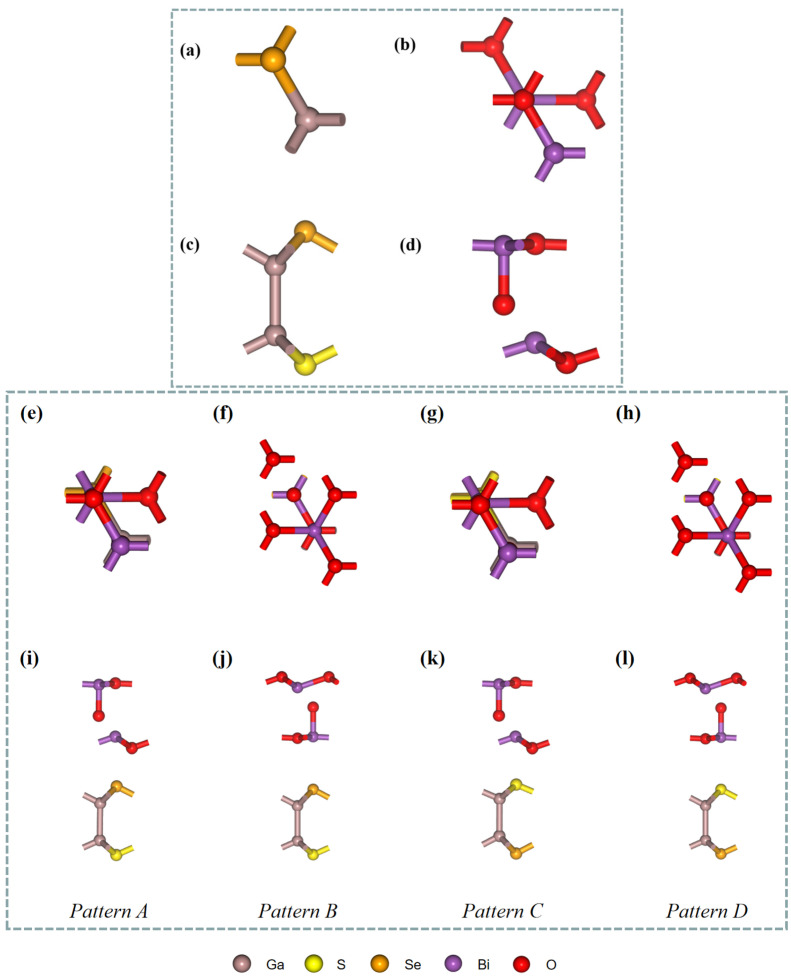
Top and side views of the unit cells of Ga_2_SSe monolayer (**a**,**c**); Bi_2_O_3_ monolayer (**b**,**d**); Janus Ga_2_SSe/Bi_2_O_3_ vdW heterojunctions, distinguished by the element type on both sides of the vdW gap as follows: Pattern A (**e**,**i**), Pattern B (**f**,**j**), Pattern C (**g**,**k**), and Pattern D (**h**,**l**).

**Figure 2:**
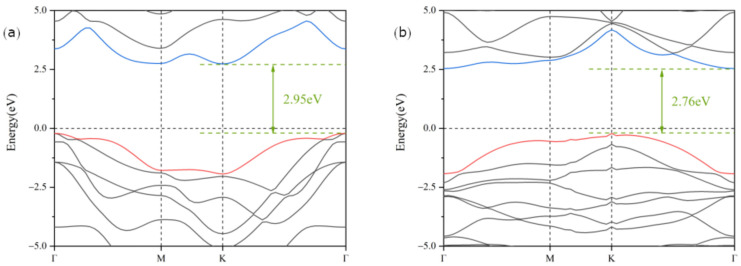
Band structures of the Ga_2_SSe (**a**) and Bi_2_O_3_ (**b**) monolayers calculated using the HSE06 functional. The Fermi level is shifted to zero.

The of Ga_2_SSe and Bi_2_O_3_ monolayers were obtained by cleaving the hexagonal Ga_2_SSe and Bi_2_O_3_ crystals along the (001) direction. The optimized structures of Ga_2_SSe and Bi_2_O_3_ are presented in Figure 1a–d. The lattice constants for Ga_2_SSe and Bi_2_O_3_ monolayers were calculated to be 3.71 Å and 3.87 Å, which align well with earlier reports [52,53].The small lattice mismatch of 4.2% between Ga_2_SSe and Bi_2_O_3_ suggests their capability to establish a stable heterojunction within the two-dimensional plane. Owing to the structural asymmetry of the Ga_2_SSe and Bi_2_O_3_ monolayers, four distinct heterojunction configurations were constructed. These configurations vary based on the chemical element positioned on either side of the van der Waals gap, as shown in Figure 1e–l, and are designated as patterns A, B, C, and D. The electronic energy band structures of Ga_2_SSe and Bi_2_O_3_ monolayers were calculated using the HSE06 hybrid functional, as shown in Figure 2a,b. The results indicate that both Ga_2_SSe and Bi_2_O_3_ monolayers are indirect bandgap semiconductors, with bandgaps of 2.95 eV and 2.76 eV. These findings align with previous reports [54,55].

The four different heterojunctions were optimized, resulting in the lattice parameters (with a = b), interfacial distances (d), and interfacial energies (E_int_) gathered in Table 1. The stability of the different heterojunctions can be estimated using the following equation:

(1) $$E_{int}=E_{{Ga}_{2}SSe/{Bi}_{2}O_{3}}-E_{{Ga}_{2}SSe}-E_{{Bi}_{2}O_{3}}$$

where E_Ga2SSe/Bi2O3_ represents the total energy of the heterojunction, while E_Ga2SSe_ and E_Bi2O3_ correspond to the total energies of the individual monolayers. Negative interfacial energies indicate stabilizing interactions between the two monolayers of the heterojunctions, and the lower the E_int_ value is, the more stable the formed heterojunction is.

The results in Table 1 suggest the feasible formation of all four heterojunctions, and that patterns A and C are more stable than patterns B and D. These two most stable patterns exhibit the smallest interlayer distances, which could be ascribed to strong interactions between oxygen and sulfur (or selenium) atoms, due to their proximity in the considered configuration (see Figure 1).

**Figure 3:**
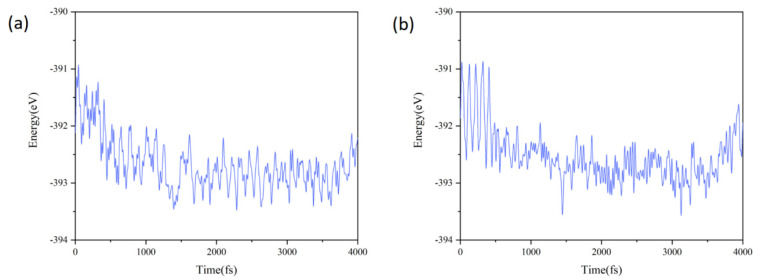
Fluctuations of the total free energy of (**a**) pattern A and (**b**) pattern C heterojunctions throughout the AIMD simulations.

The band gaps of heterojunctions B and D (0.32 eV and 0.30 eV, respectively) clearly do not meet the requirements for photocatalytic water splitting (see below for band gap values). Therefore, their thermal stability was not further analyzed using molecular dynamics simulations. In the subsequent analysis, ab initio molecular dynamics (AIMD) simulations were performed to further investigate the thermal stability of heterojunctions A and C. The simulations were conducted using a supercell configuration of 3 × 3 × 1, with the temperature fixed at 300 K. Figure 3a,b illustrate the fluctuations in the total energy of the pattern A and C heterojunctions. These figures reveal that the total energy fluctuations for both heterojunctions are significant during the initial 500 fs. However, as time extends to 4000 fs, the fluctuations stabilize at weak levels. Given this stabilization of the fluctuations and the fact that the structures of heterojunctions A and C have not been drastically modified by the end of the simulation, we can conclude that these heterojunctions are thermally stable at an ambient temperature [56,57].

### 3.2. Electronic Properties

**Figure 4:**
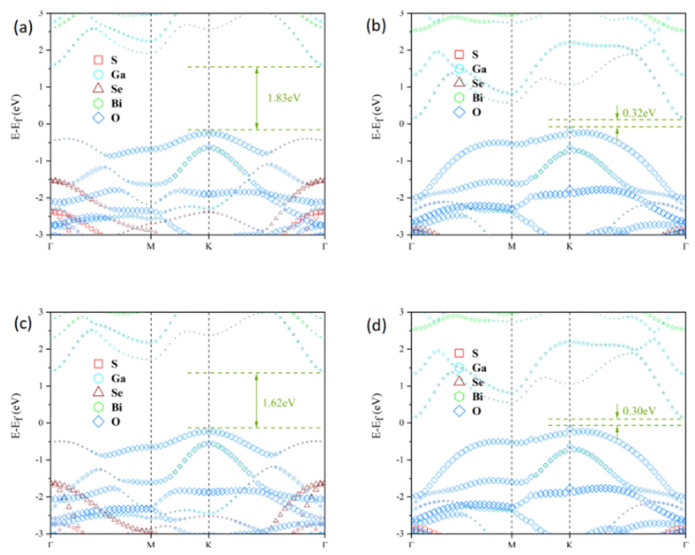
Atom-projected band structures of Janus Ga_2_SSe/Bi_2_O_3_ vdW heterojunctions computed using the HSE06 functional for pattern A (**a**), pattern B (**b**), pattern C (**c**), and pattern D (**d**), as defined in Figure 1. The Fermi energy is shifted to 0 eV.

**Figure 5:**
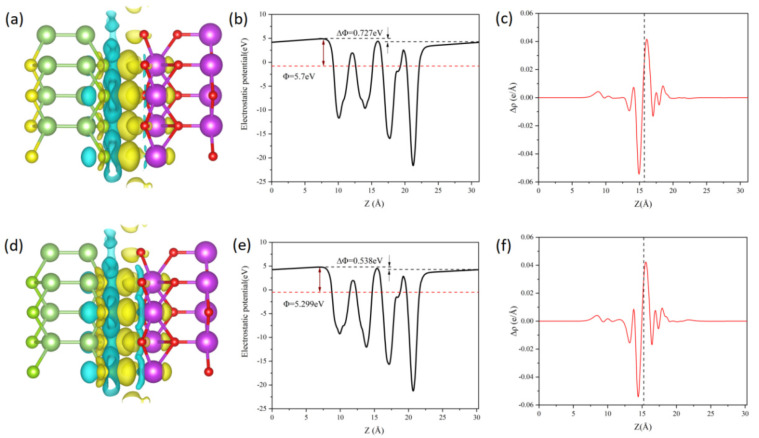
Charge density difference of Janus Ga_2_SSe/Bi_2_O_3_ vdW heterojunctions: pattern A (**a**) and pattern C (**d**). The isovalue is set to 0.0005 e/Å^3^. Electrostatic potential profiles across the interface of pattern A (**b**) and pattern C (**e**) heterojunctions. Planar-averaged electron density difference, Δρ(z), for pattern A (**c**) and pattern C (**f**) heterojunctions. Yellow and cyan regions indicate electron accumulation and depletion, respectively.

To investigate the electronic properties of the Janus Ga_2_SSe/Bi_2_O_3_ vdW heterojunctions, atom-projected band structures were computed using the HSE06 functional, as shown in Figure 4a–d. In all patterns (A–D), the valence band maximum (VBM) is positioned at the K point, whereas the conduction band minimum (CBM) appears at the Γ point. Therefore, all four heterojunction patterns exhibit indirect bandgaps, with values of 1.83 eV, 0.32 eV, 1.62 eV, and 0.30 eV, respectively. We observe that the bandgap width can be correlated to the van der Waals interlayer distance and to the nature of the atoms that are closest to each other across the vdW gap. Indeed, for the heterojunctions A and C, the closest atoms are selenium and oxygen (pattern A) and sulfur and oxygen (pattern C). These patterns lead to the smallest interlayer distances (2.533 Å and 2.396 Å) and largest bandgap widths (1.83 eV and 1.62 eV). Additionally, the larger the interlayer distance the larger the bandgap energy is. For the heterojunctions B and D, the nearest atoms across the vdW gaps are gallium and selenium (pattern B) and gallium and sulfur (pattern D). The bandgap energies are rather small and quite similar, with values 0.32 eV and 0.30 eV. Incidentally, we observe the same similarity for the vdW gap distances (3.587 Å and 3.586 Å). The smallness of the bandgap energies observed for these heterojunctions is probably related to the presence of the gallium near the vdW gaps, which provides a small metallic character to the structures. The modulation of the electronic structure of heterojunctions has already been reported in the literature [58,59,60,61]. Additional analysis demonstrates that the VBM and CBM of the four heterojunction patterns stem from the Bi_2_O_3_ and Ga_2_SSe layers, respectively. This suggests that all the heterojunctions exhibit a type-II band alignment, which effectively hinders the recombination of photogenerated electron-hole pairs. Notably, the bandgaps of pattern B and D heterojunctions do not satisfy the minimum energy threshold of 1.23 eV for photocatalytic water splitting. Therefore, the following analysis primarily focuses on the pattern A and C heterojunctions. Furthermore, the three-dimensional charge density difference (CDD) was computed to analyze charge transfer and separation at the interfaces of the pattern A and C heterojunctions, as illustrated in Figure 5a,d. The yellow regions indicate charge accumulation, whilst the cyan regions denote charge depletion. The CDD was calculated using the following equation [62]:

(2) $$\Delta \rho =\rho_{Ga_{2}SSe/Bi_{2}O_{3}}-\rho_{Ga_{2}SSe}-\rho_{Bi_{2}O_{3}}$$

where ρ_Ga2SSe/Bi2O3_, ρ_Ga2SSe_, and ρ_Bi2O3_ correspond to the total charge density of the heterojunction and the individual monolayers, respectively. Analysis of the CDD reveals that the Ga_2_SSe layer transfers electrons to the Bi_2_O_3_ layer. Bader charge analysis indicates that about 0.0343 |e| for pattern A and 0.0294 |e| for pattern C are transferred from the Ga_2_SSe layer to the Bi_2_O_3_ layer at the heterojunction interface. This indicates that the charge transfer mechanism in pattern A and C heterojunctions corresponds to that of a direct Z-scheme heterojunction. These findings resemble the electron transfer behavior reported in Janus MoSSe/Ga_2_SSe vdW heterojunctions [20].

In order to determine the direction of the electric field and charge transfer, the electrostatic potential of the pattern A and pattern C heterojunctions was analyzed along the z-axis [63]. The findings are presented in Figure 5b,e. Owing to the difference in vacuum energy levels, the pattern A and pattern C heterojunctions exhibit electrostatic potential differences of 0.727 eV and 0.538 eV, respectively. It is clear that the Bi_2_O_3_ layer possesses a lower potential than the Ga_2_SSe layer, causing electrons to migrate from the Ga_2_SSe layer to the Bi_2_O_3_ layer. This is further validated by the planar-averaged electron density difference, as depicted in Figure 5c,f. The planar-averaged electron density difference was determined by integrating the in-plane CDD, following the equation [62]:

(3) $$\Delta \rho \left(z\right)=\int \rho_{Ga_{2}SSe/Bi_{2}O_{3}}dxdy-\int \rho_{Ga_{2}SSe}dxdy-\int \rho_{Bi_{2}O_{3}}dxdy$$

where ∫ρ_Ga2SSe/Bi2O3_dxdy, ∫ρ_Ga2SSe_dxdy, and ∫ρ_Bi2O3_dxdy represent the charge densities of the heterojunction and the two monolayers, respectively, integrated over the xy plane. The transfer of electrons from the Ga_2_SSe layer to the Bi_2_O_3_ layer results in the establishment of an internal electric field directed from the Bi_2_O_3_ layer to the Ga_2_SSe layer at the heterojunction interface. This built-in electric field direction is beneficial to the separation of photogenerated electron-hole pairs.

### 3.3. Photocatalytic Water-Splitting Properties

For effective water decomposition, photocatalytic materials must meet two essential requirements: (1) a band gap exceeding 1.23 eV, and (2) redox potentials of water positioned within the band gap [64]. More precisely, the conduction band minimum (CBM) should be greater than the water reduction potential, while the valence band maximum (VBM) must be less than the oxidation potential. According to literature [65], the water reduction and oxidation potentials at pH = 0 are −5.67 eV and −4.44 eV, respectively. To evaluate the potential of the pattern A and C heterojunctions for photocatalytic water splitting, the band edge positions were determined using the following equations:

(4) $$E_{VBM}=-I=-\chi -0.5E_{g}$$

(5) $$E_{CBM}=-A=E_{VBM}+E_{g}$$

where I, A, χ, and E_g_ denote the ionization energy, electron affinity, absolute electronegativity, and band gap of the respective material, respectively. These parameters are critical for determining the VBM and CBM positions and evaluating whether the patterns A and C heterojunctions meet the necessary conditions for photocatalytic activity. Generally, conventional photocatalytic materials must possess a band gap exceeding 1.23 eV to promote water-splitting reactions. However, following a novel methodology introduced by Li et al. [66], the band gap threshold for polar materials is revised as Eg > 1.23−ΔΦ eV, where ΔΦ represents the static potential difference, with ΔΦ > 0.

The standard potential expressions for the H^+^/H_2_ and O_2_/H_2_O couples in aqueous solutions as a function of the pH can be expressed as follows [35,65]:

(6) $$E_{H^{+}/H_{2}}=-4.44+0.059pH$$

(7) $$E_{O_{2}/H_{2}O}=-5.67+0.059pH$$

**Figure 6:**
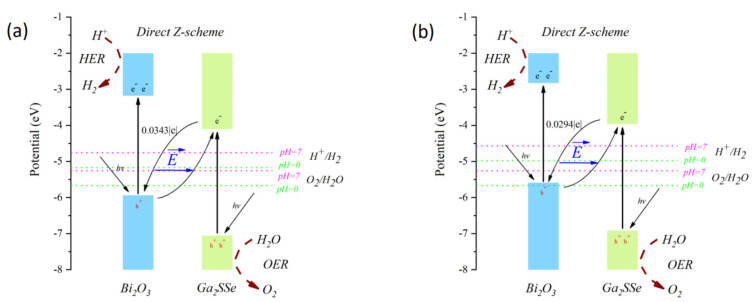
Corrected band edge positions of the Janus Ga_2_SSe/Bi_2_O_3_ vdW heterojunctions: pattern A (**a**) and pattern C (**b**) according to Li et al. [66].

At a pH of zero, the water reduction and oxidation potentials are −5.67 eV and −4.44 eV, respectively. As the pH increases to 7, these potentials shift to −5.26 eV and −4.03 eV. Based on the method outlined by Li et al. [66], the corrected band edge positions of the patterns A and C heterojunctions were calculated and are illustrated in Figure 6. For the pattern A heterojunction, the band edge position meets the water redox potentials at both pH = 0 and pH = 7. In contrast, the band edge position of the pattern C heterojunction satisfies the water redox potentials only at pH = 7. These results indicate that pattern A can promote both the oxygen evolution reaction (OER) and hydrogen evolution reaction (HER) in acidic and neutral environments, while pattern C is limited to a pH range of 1.2 to 7.

### 3.4. Optical Properties

To determine the optical properties of the Ga_2_SSe/Bi_2_O_3_ vdW heterojunctions and the isolated Ga_2_SSe and Bi_2_O_3_ monolayers, the dielectric function (ε) [67] was calculated, and the absorption coefficient (α) was derived accordingly [68]. The real and imaginary parts of ε are interdependent, with ε_2_ obtained from ε_1_ via the Kramers–Kronig transformation.

**Figure 7:**
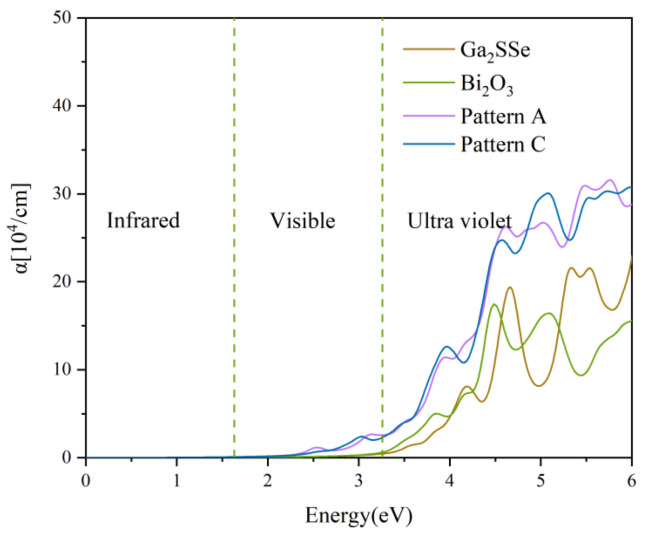
Optical absorption spectra of the Ga_2_SSe monolayer, Bi_2_O_3_ monolayer, and Ga_2_SSe/Bi_2_O_3_ vdW heterojunctions (pattern A and pattern C) calculated using the HSE06 functional. The vertical blue dotted lines delineate the boundaries of the visible wavelength region.

The absorption coefficients of the patterns A and C heterojunctions, as well as those of the isolated Ga_2_SSe and Bi_2_O_3_ monolayers, are shown in Figure 7. In the visible region, Ga_2_SSe and Bi_2_O_3_ monolayers have a very light absorption. In contrast, the absorption coefficients of the patterns A and C heterojunctions are significantly higher than those of the Ga_2_SSe and Bi_2_O_3_ monolayers across the visible and UV light spectrum. Notably, the heterojunctions exhibit markedly enhanced absorption capabilities within the visible light range, indicating that the Ga_2_SSe/Bi_2_O_3_ vdW heterojunctions are promising candidates for photocatalytic applications.

To gain deeper insight into the photocatalytic performance, the light absorption conversion efficiency (η_abs_), carrier utilization efficiency (η_cu_), solar-to-hydrogen (STH) efficiency (η_STH_), and corrected STH (η’_STH_) efficiency were calculated [69,70]. The η_abs_ value is computed using the following equation:

(8) $$\eta_{abs}=\frac{\int_{Eg}^{\infty }\;P(\hbar \omega )d(\hbar \omega )}{\int_{0}^{\infty }\;P(\hbar \omega )d(\hbar \omega )}$$

where P(ℏω) represents the AM1.5G solar energy flux at the photon energy ℏω, and E_g_ denotes the bandgap energy. The efficiency related to the carrier utilization is given by the following:

(9) $$\eta_{cu}=\frac{\Delta G\int_{E}^{\infty }\;\frac{P(\hbar \omega )}{\hbar \omega }d(\hbar \omega )}{\int_{E_{g}}^{\infty }\;P(\hbar \omega )d(\hbar \omega )}$$

where ΔG represents the energy required for the water-splitting reaction. The energy E of the photons utilized for water splitting depends on the overpotentials χ(H_2_) and χ(O_2_) for the hydrogen and oxygen evolution reactions, respectively.

(10) $$E=\left\{\begin{array}{c} E_{g},\;\;\;\;\;\;\;\;\;\;\;\;\;\;\;\;\;\;\;\;\;\;\;\;\;\;\;\;\;\;\;\;\;\;\;\;\;\;\;\;\;\;\;\;\;\;\;\;\;\;\;if\;\chi (H_{2})\geq 0.2\;\mathrm{and}\;\chi (O_{2})\geq 0.6\\ E_{g}+0.2-\chi (H_{2}),\;\;\;\;\;\;\;\;\;\;\;\;\;\;\;\;\;\;\;\;\;\;\;if\;\chi (H_{2})<0.2\;\mathrm{and}\;\chi (O_{2})\geq 0.6\\ E_{g}+0.6-\chi (O_{2}),\;\;\;\;\;\;\;\;\;\;\;\;\;\;\;\;\;\;\;\;\;\;\;\;if\;\chi (H_{2})\geq 0.2\;\mathrm{and}\;\chi (O_{2})<0.6\\ E_{g}+0.8-\chi (H_{2})-\chi (O_{2}),\;\;\;\;\;\;\;if\;\chi (H_{2})<0.2\;\mathrm{and}\;\chi (O_{2})<0.6 \end{array}\right.$$

Here, χ(H_2_) is derived from the difference between the CBM and the H^+^/H_2_ redox potential, while χ(O_2_) is calculated from the difference between the VBM and the H_2_O/O_2_ redox potential. The STH efficiency is subsequently computed using the following equation:

(11) $$\eta_{STH}=\eta_{abs}\times \eta_{cu}$$

Considering the beneficial role played by the intrinsic electric field on the separation of electrons and holes, η_STH_ must be adjusted as follows:

(12) $$\eta_{STH}^{’}=\eta_{STH}\times \frac{\int_{0}^{\infty }\;P(\hbar \omega )d(\hbar \omega )}{\int_{0}^{\infty }\;P(\hbar \omega )d(\hbar \omega )+\Delta \Phi \int_{E_{g}}^{\infty }\;\frac{P(\hbar \omega )}{\hbar \omega }d(\hbar \omega )}$$

where ΔΦ indicates the difference in vacuum energy levels between the upper and lower surfaces. The calculated efficiencies are summarized in Table 2. The correction has a minimal impact on the STH efficiency; however, the corrected STH efficiency values of the patterns A and C heterojunctions are 13.60% and 12.08%, respectively. These values are of the same order of magnitude as the commercial benchmark (10%) [71]. In contrast, the STH efficiencies of the bilayer Ga_2_SSe vdW homojunction [72], GaSe/CN vdW heterojunction [73], GaN/Ga_2_SSe heterojunction [74], and Cu_2_Se/SIn_2_S vdW heterojunction [75] are 6.49%, 11.56%, 10.62%, and 14.35%, respectively. These results further suggest that Ga_2_SSe/Bi_2_O_3_ vdW heterojunctions have significant potential for practical implementation in the future.

**Figure 8:**
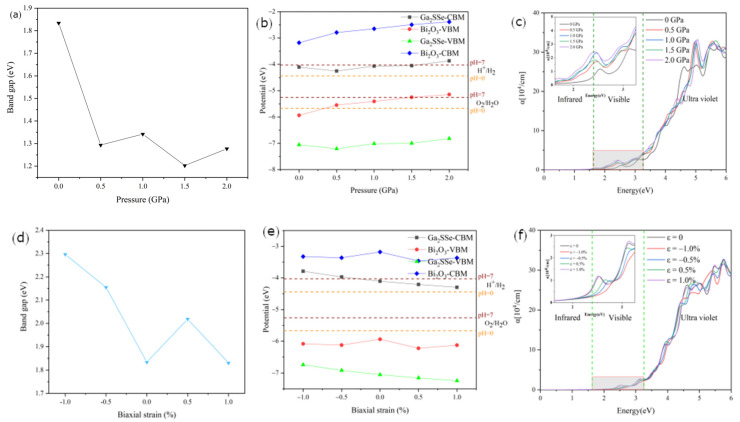
(**a**) Band gap, (**b**) band edge positions, and (**c**) absorption spectra of the Ga_2_SSe/Bi_2_O_3_ (pattern A) vdW heterojunction under different pressures. (**d**) Band gap, (**e**) band edge positions, and (**f**) absorption spectra of the Ga_2_SSe/Bi_2_O_3_ (pattern A) vdW heterojunction under biaxial strain. The insets in (**c**,**f**) feature an enlarged view of the visible region.

We further investigated the effects of pressure and strain on the electronic and optical properties of the Ga_2_SSe/Bi_2_O_3_ pattern A vdW heterojunction, which exhibits the highest corrected solar-to-hydrogen efficiency. Through a systematic application of hydrostatic pressure (Figure 8a), we observed that the bandgap of the heterojunction decreased under increasing pressure, stabilizing at values around 1.30 eV. Without considering the electrostatic potential difference (ΔΦ), the corresponding band edge positions at different hydrostatic pressures are presented in Figure 8b. In this Z-scheme configuration, the OER occurs at the VBM of Ga_2_SSe, while the HER takes place at the CBM of Bi_2_O_3_. Under increasing hydrostatic pressure, the CBM of Bi_2_O_3_ shows a gradual upward shift, consistently exceeding the oxidation potential of water at a pH of 7, thereby promoting the HER to a certain extent. Conversely, the VBM of Ga_2_SSe exhibits minor fluctuations but remains below the water reduction potential. At pressures up to 2.0 GPa, the OER is relatively weakened. Furthermore, we analyzed the pressure-dependent optical absorption characteristics of the heterojunction (Figure 8c), revealing an enhanced absorption efficiency extending from the UV to visible regions as hydrostatic pressure increases.

Previous studies have established that strain engineering is an effective approach for modulating the electronic properties of heterojunctions [76,77]. In the present work, we investigated the strain-dependent electronic properties of the pattern A heterojunction by precisely controlling the lattice parameters and applying biaxial strain ranging from −1% to +1%. The strain (ε) was calculated using the following equation:

(13) $$\varepsilon =\frac{b-b_{0}}{b_{0}}\times 100\%$$

where b and b_0_ represent the lattice parameters with and without strain, respectively. A positive ε value indicates tensile strain in the heterojunction, while a negative ε value corresponds to compressive strain.

Figure 8d illustrates the strain-dependent evolution of the bandgap in the heterojunction under biaxial strain. The bandgap demonstrates a significant increase with increasing compressive strain, while under tensile strain, it initially increases before decreasing—a phenomenon attributed to modulated interlayer orbital interactions. As depicted in Figure 8e, the CBM of Bi_2_O_3_ exhibits minor fluctuations but consistently remains above the water reduction potential throughout the transition from compression to tension. Concurrently, the VBM of Ga_2_SSe displays a decreasing trend, effectively enhancing the OER activity. Furthermore, we systematically evaluated the influence of biaxial strain on the optical absorption properties of the heterojunction. As shown in Figure 8f, the heterojunction demonstrates enhanced visible light absorption at 1% tensile strain, indicating improved solar energy harvesting efficiency.

## 4. Conclusions

In this study, we systematically investigated the structural stability, electronic structures, and optical properties of the Ga_2_SSe/Bi_2_O_3_ vdW heterojunction and their potential use in water splitting, through first-principles calculations. Both the interfacial energy (E_int_) calculations and AIMD simulations confirm the stability of the Ga_2_SSe/Bi_2_O_3_ heterojunctions formed between the Ga_2_SSe and Bi_2_O_3_ layers. Among the four considered pattern (A, B, C, and D) heterojunctions, only pattern A and C heterojunctions satisfy the bandgap requirements for photocatalytic water splitting, with gap values of 1.83 eV and 1.62 eV, respectively. Additionally, they are both direct Z-scheme photocatalysts according to the electronic properties and Bader charge analyses. The Bi_2_O_3_ and Ga_2_SSe layers serve as photocatalysts for HER and OER separately, with strong redox capability for water splitting into hydrogen and oxygen. Charge transfer at the heterojunction interface generates a built-in electric field directed from Bi_2_O_3_ to Ga_2_SSe, favoring electron-hole recombination across the heterojunction and hindering reverse charge carrier transfer, thereby enhancing photoinduced electron-hole pair separation efficiency. Compared with the Ga_2_SSe and Bi_2_O_3_ monolayers, patterns A and C heterojunctions exhibit higher light absorption intensities across the visible to ultraviolet spectrum, with corrected solar-to-hydrogen efficiency (η’S_TH_) values of 13.60% and 12.08%, respectively. We systematically studied the effects of hydrostatic pressure and biaxial tensile strain on the photocatalytic performance of the pattern A heterojunction. Hydrostatic pressure preferentially enhances HER activity, while biaxial tensile strain significantly improves OER efficiency. The light absorption properties of the heterojunction are enhanced under both hydrostatic pressure and 1% biaxial strain, especially in the visible spectral region. Therefore, the Ga_2_SSe/Bi_2_O_3_ heterojunctions are highly promising photocatalysts with great potential and wide applications for water splitting.

## Figures and Tables

**Table 1:** Calculated values for lattice parameters (a), interlayer distances (d), and interfacial energies (E_int_) for the four configurations of Janus Ga_2_SSe/Bi_2_O_3_ vdW heterojunctions.

Pattern	a (Å)	d (Å)	E_int_ (meV)
A	3.829	2.533	−108.5
B	3.815	3.587	−19.5
C	3.831	2.396	−120.6
D	3.815	3.586	−15.7

**Table 2:** Overpotentials for hydrogen and oxygen evolution reactions (χ(H_2_) and χ(O_2_) at pH = 7, in eV), energy conversion efficiencies of light absorption (η_abs_), carrier utilization (η_cu_), solar-to-hydrogen (STH) (η_STH_), and corrected STH (η’_STH_) of Janus Ga_2_SSe/Bi_2_O_3_ (patterns A and C) vdW heterojunctions.

Heterojunction	χ(H_2_)	χ(O_2_)	η_abs_ (%)	η_cu_ (%)	η_STH_ (%)	η’_STH_ (%)
Pattern A	0.65	0.68	27.68	53.45	14.79	13.60
Pattern C	0.60	0.33	36.30	36.34	13.19	12.08

## Data Availability

Data are available upon request.

## Abbreviations

The following abbreviations are used in this manuscript:

DFT	Density functional theory
vdW	Van der Waals
STH	Solar-to-hydrogen
TMCs	Transition metal chalcogenides
VASP	Vienna Ab initio Simulation Package
PBE	Perdew–Burke–Ernzerhof
GGA	Generalized gradient approximation
PAW	Projector-augmented waves
HSE06	Heyd–Scuseria–Ernzherof
AIMD	Ab initio molecular dynamics
VBM	Valence band maximum
CBM	Conduction band minimum
CDD	Charge density difference
OER	Oxygen evolution reaction
HER	Hydrogen evolution reaction
